# Screening and Application of Highly Efficient Rhizobia for Leguminous Green Manure *Astragalus sinicus* in Lyophilized Inoculants and Seed Coating

**DOI:** 10.3390/plants14152431

**Published:** 2025-08-06

**Authors:** Ding-Yuan Xue, Wen-Feng Chen, Guo-Ping Yang, You-Guo Li, Jun-Jie Zhang

**Affiliations:** 1 College of Biological Sciences and Rhizobium Research Center, China Agricultural University, Beijing 100193, China; bs20233020217@cau.edu.cn; 2 Ningxia Wu Feng Agricultural Technology Co., Ltd., Yinchuan 750021, China; 3 National Key Laboratory of Agricultural Microbiology, College of Life Science and Technology, Huazhong Agricultural University, Wuhan 430070, China; youguoli@mail.hzau.edu.cn; 4 College of Food and Bioengineering, Zhengzhou University of Light Industry, Zhengzhou 450002, China; junjiezh@zzuli.edu.cn

**Keywords:** nitrogen-fixing efficacy, cryoprotectant formulation, bacterial viability, nodulation capacity, tiller number, aboveground biomass, chlorophyll content, seed pelleting, microbial survival, sustainable rice rotation

## Abstract

*Astragalus sinicus*, a key leguminous green manure widely cultivated in Southern China’s rice-based cropping systems, plays a pivotal role in sustainable agriculture by enhancing soil organic matter sequestration, improving rice yield, and elevating grain quality. The symbiotic nitrogen-fixing association between *A. sinicus* and its matching rhizobia is fundamental to its agronomic value; however, suboptimal inoculant efficiency and field application methodologies constrain its full potential. To address these limitations, we conducted a multi-phase study involving (1) rhizobial strain screening under controlled greenhouse conditions, (2) an optimized lyophilization protocol evaluating cryoprotectant (trehalose, skimmed milk powder and others), and (3) seed pelleting trails with rhizobial viability and nodulation assessments over different storage periods. Our results demonstrate that *Mesorhizobium huakuii* CCBAU 33470 exhibits a superior nitrogen-fixing efficacy, significantly enhancing key traits in *A. sinicus*, including leaf chlorophyll content, tiller number, and aboveground biomass. Lyophilized inoculants prepared with cryoprotectants (20% trehalose or 20% skimmed milk powder) maintained >90% bacterial viability for 60 days and markedly improved nodulation capacity relative to unprotected formulations. The optimized seed pellets sustained high rhizobial loads (5.5 × 10^3^ cells/seed) with an undiminished viability after 15 days of storage and nodulation ability after 40 days of storage. This integrated approach of rhizobial selection, inoculant formulation, and seed coating overcomes cultivation bottlenecks, boosting symbiotic nitrogen fixation for *A. sinicus* cultivation.

## 1. Introduction

*Astragalus sinicus* L. (Chinese milk vetch), native to China, is a widely cultivated leguminous green manure crop, primarily grown in rice-based cropping systems across Southern China, with major distributions in the provinces of Anhui, Zhejiang, Hunan, Hubei, Henan, and Fujian [1], as well as Japan [2]. Renowned for its high nitrogen-fixing efficiency and adaptability to humid environments [3], *A. sinicus* delivers multiple agronomic benefits, including increased rice yields, improved grain quality, promoted nutrient usage efficiency, and enhanced soil fertility through organic matter accumulation [3,4,5,6]. Long-term cultivation in rice—*A. sinicus* rotations significantly elevates soil organic matter content while providing ecological benefits such as weed suppression [7], improved soil iron nutrition [8], and mitigation of cadmium contamination [9]. Reducing nitrogen input enhances symbiotic nitrogen fixation in *A. sinicus*, improving nodulation, nitrogenase activity, and *nifH* expression, thereby contributing to improved rice growth in rotational cropping systems [10].

The plant’s nitrogen-fixing capability stems from its symbiotic relationship with three *Mesorhizobium* species: *M. huakuii* (predominantly in southeastern China and Japan) [11,12], *M. qingshengii* (Jiangxi Province) [2], and *M. jarvisii* (Henan Province) [13]. While compatible rhizobia strains can increase host plant yield by ≥15% [11], current inoculation techniques face limitations in efficiency and field application. Conventional methods (seed coating, peat-based pellet inoculants, and furrow spray) [14] often suffer from poor rhizobial survival during storage and field application. Although lyophilization with cryoprotectants including carbohydrates (e.g., trehalose, sucrose, mannitol), proteins (e.g., skimmed milk powder, bovine serum albumin, gelatin), and polymers (e.g., polyethylene glycol, polyvinylpyrrolidone) can enhance microbial preservation [15,16,17,18], optimal formations for *A. sinicus* rhizobia remain underexplored. Similarly, while seed coating technology offers operational advantages [19,20,21], existing protocols lack integration with rhizobial inoculant for seeds of *A. sinicus*.

Building on the host-specific symbiosis between *A. sinicus* and *Mesorhizobium* spp. [1,2,11], we hypothesized the following: (1) screening rhizobial strains with high nitrogen-fixing efficiency would enhance *A. sinicus* productivity; (2) lyophilization with optimized cryoprotectants would improve rhizobial viability in inoculants [15,19,20,21]; (3) seed pelleting with rhizobia would maintain microbial viability and nodulation ability during storage before field applicability.

To test these hypotheses, we pursue three objectives in this study: (i) Systematic screening of *Mesorhizobium* strains for elite *A. sinicus* symbionts; (ii) Development of optimized lyophilized formulations with long-term viability; (iii) Engineering of seed pellets integrating rhizobia and seed coating materials. This integrated approach overcomes key limitations in inoculant technology and introduces an innovative strategy for strain-specific rhizobium through rhizobia-pelleted *A. sinicus* seeds in sustainable rice-based rotation systems.

## 2. Results

### 2.1. Screening and Evaluation of High-Efficiency Nitrogen-Fixing Mesorhizobium Strains for A. sinicus

**Figure 1:**
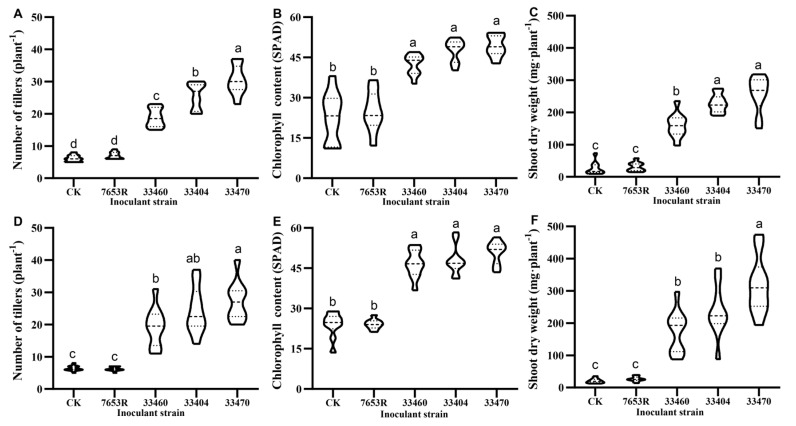
Comparative growth performance of two *A. sinicus* varieties inoculated with various rhizobial strains at 45 days post-inoculation. Upper panels show the tiller number (**A**), chlorophyll content (SPAD value, (**B**)), and aboveground biomass (**C**) for variety Luzi No. 8, while lower panels (**D**–**F**) show the corresponding parameters for variety Yijiangzi. Different lowercase letters above bars indicate statistically significant differences (*p* < 0.05, one-way ANOVA).

We conducted a systematic screening of rhizobial strains to determine their symbiotic efficiency with *A. sinicus* by evaluating multiple nodulation-capable isolates from our rhizobial strain library (CCBAU, Beijing, China). The results of greenhouse experiments involving two *A. sinicus* cultivars (Luzi No. 8 and Yijiangzi) inoculated with four *Mesorhizobium* strains (CCBAU 33404, CCBAU 33470, CCBAU 33460, and 7653R) revealed significant strain-dependent variation in plant growth promotion after 45 days (Supplementary Figures S1 and S2). While CCBAU 7653R showed comparable performance to uninoculated controls, strains CCBAU 33404, CCBAU 33470, and CCBAU 33460 significantly enhanced plant growth parameters, including tiller number, leaf chlorophyll content (SPAD values), and aboveground biomass (Figure 1).

Notably, strain CCBAU 33470 demonstrated superior performance, inducing the highest tiller production in both cultivars (Figure 1A,D), which correlated with improved plant vigor given *A. sinicus*’s characteristic clustered growth habit featuring slender stems and nutrient-dependent tiller development. These results position strain CCBAU 33470 as a particularly promising candidate for enhancing *A. sinicus* productivity through optimized nitrogen fixation.

In the Luzi No. 8 cultivar, inoculation with strain CCBAU 33470 significantly increased tiller number to 30.40 ± 4.58 per plant, representing a 4.98-fold enhancement over the control (CK). This strain outperformed both strains CCBAU 33404 (4.19 × CK) and CCBAU 33460 (3.08 × CK). In contrast, inoculation with strain 7653R (7.00 ± 1.12 tillers/plant) results in no significant difference from CK. Similarly, in the Yijiangzi cultivar, strain CCBAU 33470 induced the highest tiller production (27.30 ± 6.11 per plant, 4.27 × CK), surpassing strains CCBAU 33404 (3.80 × CK) and CCBAU 33460 (3.01 × CK). As observed in the Luzi No. 8 cultivar, again, strain 7653R (6.22 ± 0.67 tillers/plant) did not significantly differ from the control.

The results of leaf chlorophyll content analysis (Figure 1B,E) revealed substantial improvements in photosynthetic capacity following rhizobial inoculation. In Luzi No. 8, strain CCBAU 33470 inoculation elevated chlorophyll content by 116.9% relative to the control (CK), outperforming both strains CCBAU 33404 (+110%) and CCBAU 33460 (+86.6%). Similarly, in Yijiangzi, strain CCBAU 33470 demonstrated the greatest enhancement (112.1% increase vs. CK), followed by strains CCBAU 33404 (+100%) and CCBAU 33460 (+94.0%). These results further corroborate the superior symbiotic efficacy of strain CCBAU 33470 across both cultivars.

As shown in Figure 1C,F, aboveground biomass accumulation varied significantly among the inoculation treatments. The three high-efficiency strains—CCBAU 33470, CCBAU 33404, and CCBAU 33460—all significantly enhanced the dry matter production of *A. sinicus* compared to the uninoculated controls (*p* < 0.05). Notably, strain CCBAU 33470 consistently showed the strongest growth promotion effect across both cultivars, providing further evidence of its superior symbiotic performance observed in tiller number and chlorophyll content measurements.

In the formal experiment, six rhizobial strains were selected from three rhizobial species (*M. huakuii*, *M. qingshengii*, and *M. jarvisii*) capable of nodulating with *A. sinicus* for symbiotic compatibility screening tests with the Yijiangzi variety. Among them, inoculation with CCBAU 2609 and CCBAU 7653R showed no significant differences compared with the control (CK), while inoculation with CCBAU 33443, CCBAU 33460, CCBAU 33430, and CCBAU 33470 significantly promoted plant growth compared with CK (Supplementary Figure S3).

**Figure 2:**
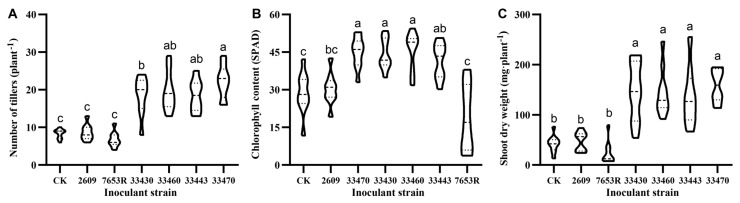
Growth responses of *A. sinicus* (variety Yijiangzi) 45 days after inoculation with different rhizobial strains. The panels illustrate the tiller number (**A**), chlorophyll content (**B**), and aboveground dry weight (**C**), respectively, of *A. sinicus*. Different lowercase letters above bars indicate statistically significant differences (*p* < 0.05, one-way ANOVA).

In the statistics of tiller numbers, we found that compared with CK, the tiller number only increased by 4.45% after inoculation with CCBAU 2609; in comparison, inoculation with 7653R decreased the tiller number by approximately 18.34%, with no significant differences among the three strains (Figure 2A). When inoculated with CCBAU 33430, the tiller number of *A. sinicus* significantly increased by 122.12%, showing a very obvious growth-promoting phenotype. When inoculated with CCBAU 33460, CCBAU 33443, and CCBAU 33470, the tiller numbers increased by 135.54%, 122.89%, and 166.39%, respectively.

Subsequently, we measured the chlorophyll content of the different treatments (Figure 2B). After inoculation with CCBAU 2609, the chlorophyll content of the plants only increased by 5.50%, and the chlorophyll content of the treatment inoculated with 7653R was also low. However, when we included the treatments inoculated with CCBAU 33430, CCBAU 33460, CCBAU 33443, and CCBAU 33470, we found that the chlorophyll content increased by 53.62%, 58.89%, 44.18%, and 56.90%, respectively.

As leguminous green manure, higher biomass in the *A. sinicus*—rice rotation system can provide more organic matter and nutrients for rice growth; as forage, high biomass results in stronger forage supply capacity. We measured the aboveground dry weight of *A. sinicus* under different treatment conditions, and the results showed that plants inoculated with CCBAU 33430, CCBAU 33460, CCBAU 33443, and CCBAU 33470 all exhibited significantly higher aboveground biomass, with increases of 230.73%, 247.81%, 220.75%, and 264.00%, respectively, compared to the control (CK) (Figure 2C), all significantly enhancing *A. sinicus* biomass. In contrast, inoculation with CCBAU 2609 and 7653R did not result in increases in aboveground dry weight.

The above results demonstrate the specific effects of the different strains on the growth of *A. sinicus*. Among them, strains CCBAU 33430, CCBAU 33460, CCBAU 33443, and CCBAU 33470 all exhibited superior performance during symbiosis with *A. sinicus*. Notably, CCBAU 33470 exhibited better effects than the other three strains. Therefore, CCBAU 33470 was selected as the representative strain for subsequent experiments.

### 2.2. Optimization of Cryoprotectants for Enhanced Rhizobial Viability

**Figure 3:**
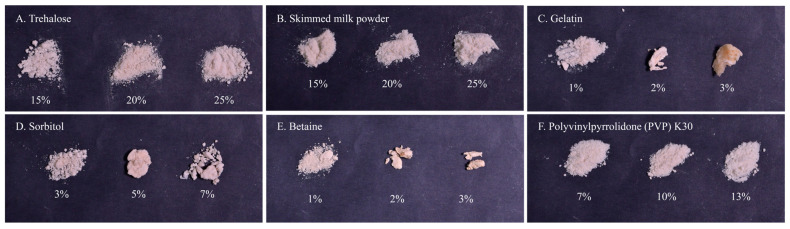
Morphological characteristics of rhizobial freeze-dried powders prepared with different cryoprotectants (**A**–**F**) and concentrations.

Comparative evaluation of six cryoprotectants (trehalose, skimmed milk powder, polyvinylpyrrolidone [PVP], gelatin, sorbitol, and betaine) revealed distinct morphological variations in the freeze–dried rhizobial formulation (Figure 3).

Visual inspection of lyophilized samples showed that trehalose-, skimmed milk powder-, and PVP-treated preparations maintained optimal physical properties, forming homogeneous, free-flowing powders (Figure 3). Conversely, gelatin-, sorbitol-, and betaine-containing samples developed increasing viscosity with higher concentrations, resulting in aggregated products unsuitable for powder processing (Figure 3).

Viable cell counts of rhizobia varied significantly among the cryoprotectant treatments (Table 1). Samples protected with 7% PVP maintained 8.7 × 10^10^ CFU/g; in comparison, 5% sorbitol yielded higher counts of 5.37 × 10^11^ CFU/g. The 20% trehalose and 20% skimmed milk powder treatments resulted in viability levels of 3.55 × 10^11^ CFU/g and 3.87 × 10^11^ CFU/g, respectively. These two formulations also exhibited favorable physical characteristics for powder processing.

**Figure 4:**
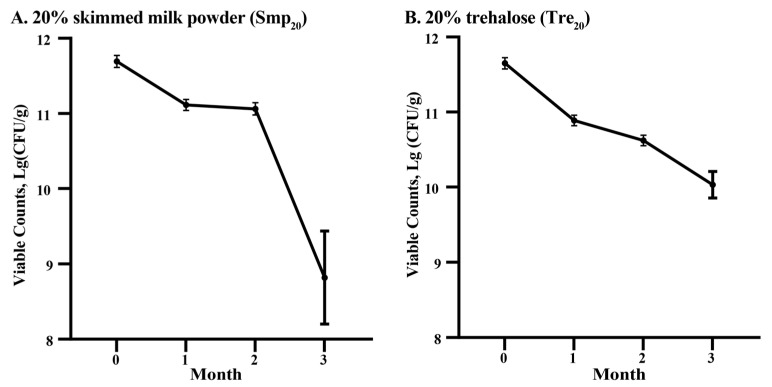
Viability of freeze-dried rhizobial formulations during 4 °C storage. Powder formulations were prepared using either (**A**) 20% skimmed milk powder (Smp_20_) or (**B**) 20% trehalose (Tre_20_) as cryoprotectants.

Viability measurements of microbial powders protected with 20% skimmed milk powder (Smp_20_) or 20% trehalose (Tre_20_) were conducted over a three-month period (Figure 4). Smp samples maintained 1.31 × 10^11^ CFU/g at month 1 and 1.17 × 10^11^ CFU/g at month 2, before declining to 1.47 × 10^9^ CFU/g at month 3 (Figure 4A). Tre samples exhibited viable counts of 7.84 × 10^10^ CFU/g at month 1, decreasing to 4.24 × 10^10^ CFU/g by month 2 and recovering to 1.16 × 10^11^ CFU/g at month 3 (Figure 4B). Both formulations maintained viability above 10^10^ CFU/g during the first two months of storage (Figure 4).

**Figure 5:**
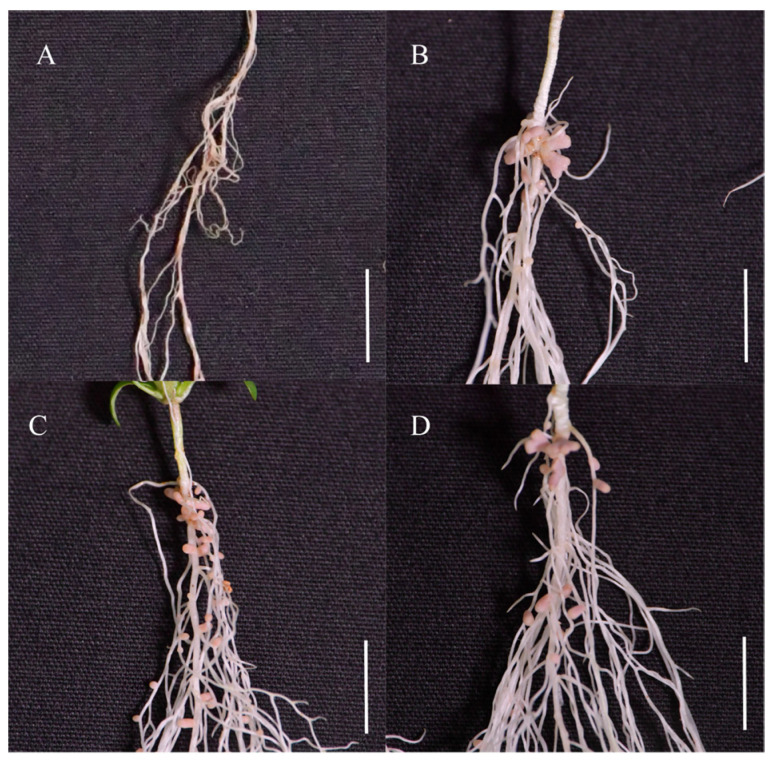
Nodulation phenotypes of *A. sinicus* (variety Yijiangzi) roots at 21 days post-inoculation with freeze-dried rhizobial powders prepared using different cryoprotectants: 20% skimmed milk powder (Smp_20_) or 20% trehalose (Tre_20_). Representative images show uninoculated control (CK, (**A**)), Tre_20_ treated root (**B**), strain CCBAU 33470 inoculated root (**C**), and Smp_20_-treated root (**D**). Bar, 1 cm.

**Figure 6:**
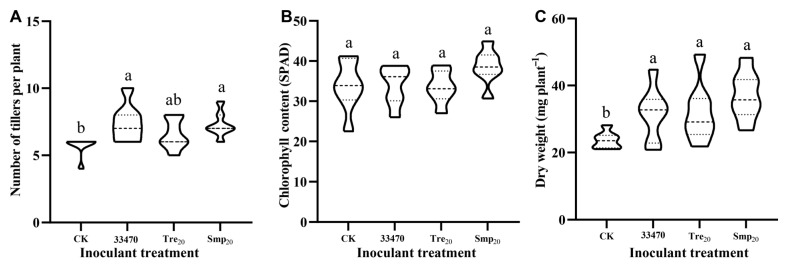
Growth parameters of *A. sinicus* (variety Yijiangzi) seedlings 21 days after inoculant treatment. Experimental groups included: uninoculated control (CK), fresh rhizobial inoculant (strain CCBAU 33470), and freeze-dried rhizobial powders containing either 20% trehalose (Tre_20_) or 20% skimmed milk powder (Smp_20_) as cryoprotectants. Measured plant parameters include: (**A**) tiller number per plant, (**B**) chlorophyll content (SPAD value) and (**C**) aboveground dry weight per plant. Different lowercase letters indicate significant differences among treatments (*p* < 0.05, one-way ANOVA).

Inoculation with Tre_20_ and Smp_20_ formulations and liquid inoculant of strain CCBAU 33470 (log-phase) resulted in the formation of characteristic nitrogen-fixing nodules on *A. sinicus* roots (Figure 5). Quantitative measurements of plant growth parameters revealed increased dry weight in both treatment groups relative to the uninoculated controls (Figure 6). Smp_20_ treatment additionally resulted in enhanced tiller production compared to Tre_20_ treatment.

### 2.3. Seed Coating Characteristics and Rhizobial Viability Assessment

**Figure 7:**
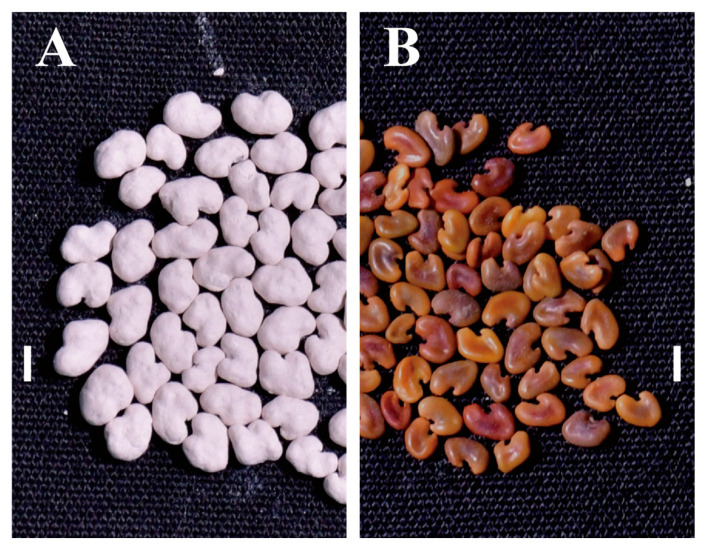
Morphological comparison between rhizobial inoculant-pelleted *A. sinicus* seeds (**A**) and the untreated ones (**B**). Bar, 2 mm.

Using a model KRT-300 pelleting machine and integration of rhizobia and coating matrix, uniformly coated *A. sinicus* seeds were produced that maintained their characteristic kidney shape while being approximately twice the size (2–3.5 mm) and weight of the uncoated seeds (Figure 7A,B). The coating process resulted in seeds with enhanced dimensional uniformity, increased thickness, complete surface coverage, and a smooth light gray appearance (Figure 7A). After drying with forced air at 25 °C, a temperature optimized for both moisture removal and rhizobial viability preservation, the coated seeds exhibited strong adhesion integrity (Figure 7A), exhibiting no detectable coating shedding under slight manual compression. When immersed in water for several minutes, the coating layer absorbed moisture and swelled, resulting in the dark brown *A. sinicus* seeds inside being exposed with gentle squeezing.

**Figure 8:**
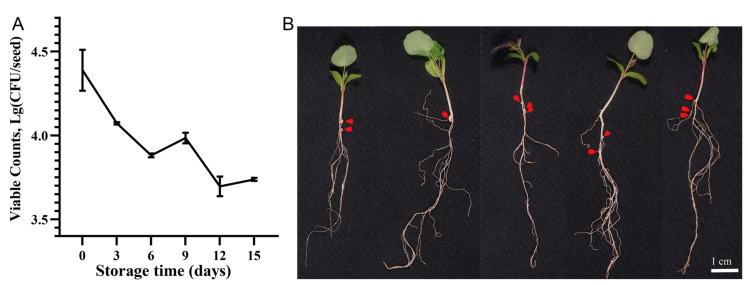
Dynamics of viable rhizobia in pelleted *A. sinicus* seeds during 15-day storage (**A**) and nodulation assessment of stored pellets after 15 days of plant growth (**B**). Red arrows indicate effective root nodules on *A. sinicus* roots (**B**).

The quantification of rhizobia inside the pelleted *A. sinicus* seeds revealed an initial bacterial load of 2.5 × 10^4^ CFU/seed immediately after coating. Following vacuum-sealed storage at 4 °C, the rhizobial population showed 52.8% viability retention (1.18 × 10^4^ CFU/seed) after four days. Bacterial viability was further monitored at three-day intervals. After 15 days of storage, approximately 5.5 × 10^3^ CFU/seed can still be detected (Figure 8A). Even after 40 days of storage, the pelleted seeds retained nodulation-competent, as indicated by the development of effective red nodules on *A. sinicus* roots and associated dark-green leaves within 15 days of growth (Figure 8B).

## 3. Discussion

### 3.1. Symbiotic Efficiency and Environmental Adaptation of Mesorhizobium Strains in A. sinicus

The establishment of effective rhizobial symbiosis is crucial for enhancing the productivity of leguminous green manure *A. sinicus*, as it directly influences nitrogen acquisition and plant growth [10,22,23,24,25,26,27,28,29,30]. Our screening of rhizobial strains with superior symbiotic compatibility—based on chlorophyll content, tiller number, and aboveground biomass—revealed significant strain-specific effects on host plant performance. Under nitrogen-limiting conditions, one highly compatible rhizobium (e.g., *M*. *huakuii* CCBAU 33470) promoted robust growth-promoting on *A. sinicus*, underscoring the importance of strain selection for optimizing nitrogen fixation efficiency [31].

Notably, symbiotic outcomes are context-dependent, influenced by soil properties and climatic factors [32,33]. This environmental plasticity suggests that regionally adapted strains, such as the widely distributed *M. huakuii* [12,34,35], may offer broader agronomic applicability. For commercial inoculant development, prioritizing strains with both high symbiotic efficiency and environmental resilience could maximize field performance across diverse cultivation conditions [36].

### 3.2. Viability Preservation of Rhizobia: Effects of Cryoprotectant and Storage

Microbial viability preservation during lyophilization presents significant challenges, primarily due to ice crystal-induced cellular damage [37]. The formation of extracellular ice crystals during freezing generates substantial osmotic stress across cellular membranes, leading to deleterious dehydration effects [38]. Furthermore, intracellular ice crystallization can cause direct mechanical disruption of cellular structures [38]. Additional viability losses may occur during sample rehydration, as the thawing process can induce secondary membrane damage [38].

Cryoprotectants employed in lyophilization protocols can be fundamentally categorized into two distinct classes based on their cellular permeability characteristics [39]. Permeable cryoprotectants, including dimethyl sulfoxide (DMSO), glycerol, and betaine (as utilized in the current study), are characterized by their ability to traverse cellular membranes [40,41]. In contrast, non-permeable protectants encompass both polymeric compounds (e.g., polyvinylpyrrolidone and skimmed milk powder, as implemented in this investigation) and low-molecular-weight carbohydrates (e.g., trehalose, sucrose, and mannitol) [15], which remain extracellular throughout the preservation process.

The viability of freeze-dried bacterial preparations is influenced by multiple critical factors throughout the culture, preservation, and resuscitation processes [42]. To optimize bacterial survival and functionality, we implemented several strategic approaches based on established cryopreservation principles. First, we extended the culture duration to ensure bacterial harvest during the stationary growth phase. This approach is supported by substantial evidence demonstrating that stationary-phase cells exhibit significantly enhanced resistance to lyophilization-induced stress compared to their logarithmic-phase counterparts [43,44]. The improved survival rates may be attributed to the accumulation of stress-response proteins and compatible solutes [45,46], in addition to the more robust cell wall structure characteristic of mature bacterial cells.

For the resuscitation process, we employed a specialized YM liquid medium supplemented with trehalose, capitalizing on its well-documented osmoprotective properties. Trehalose functions through multiple protective mechanisms, as follows: (1) forming a stabilizing glassy matrix during drying [15], (2) replacing water molecules to maintain membrane integrity [47], and (3) acting as a chemical chaperone during rehydration [45]. This optimized resuscitation protocol was designed to minimize osmotic shock and maximize the recovery of viable, metabolically active rhizobial cells while preserving their native physiological state.

The long-term viability of freeze-dried bacterial preparations is critically dependent on storage conditions, with temperature and moisture exposure representing key determinants of microbial survival [48]. The thermodynamic principles governing microbial preservation indicate that elevated temperatures accelerate molecular mobility, particularly of residual water molecules, thereby promoting deleterious chemical and physical processes, including oxidation and membrane phase transitions [37,49]. This phenomenon is well-documented in the literature, with studies demonstrating marked differences in survival rates for *Lactobacillus reuteri* maintained at 4 °C (100% viability after 4 weeks) versus 30 °C (90.0–94.8% viability) under protective conditions [50].

The detrimental effects of environmental humidity on preserved microorganisms have been extensively characterized [49,51]. Research on *Escherichia coli* has shown that exposure to humid atmospheres significantly compromises cell viability; in comparison maintenance under anhydrous conditions substantially improves survival rates [52]. This observation can be attributed to the critical relationship between water activity and cellular stability, whereby reduced moisture content minimizes hydrolytic degradation and prevents the recrystallization of amorphous matrices. In light of these established principles and as part of our preservation protocol, we incorporated two essential safeguards: (1) vacuum-sealed packaging to eliminate oxidative damage and maintain an anhydrous environment and (2) refrigeration at 4 °C to minimize thermal degradation. These measures collectively address the primary mechanisms of viability loss in lyophilized preparations, namely, oxidative stress, hydrolytic damage, and thermal denaturation. The implementation of such controlled storage conditions is particularly crucial for maintaining the metabolic competence and symbiotic capacity of rhizobial inoculants during extended storage periods prior to agricultural application.

The selection of appropriate cryoprotectants is critical for maintaining bacterial viability during lyophilization, as they mitigate cellular damage caused by ice crystal formation and dehydration stress [15,38]. Effective protectants not only preserve metabolic activity but also ensure the physical stability of freeze-dried formulations [53]. Our results demonstrate that 20% trehalose or 20% skimmed milk powder is an optimal cryoprotectant for *M. huakuii* CCBAU 33470, ensuring high viability and powder stability. While sorbitol and betaine maintained strong rhizobial activity, their physical properties limited their suitability for solid formulations, suggesting potential for liquid inoculation systems instead. These findings highlight the importance of selecting protectants based on both biological efficacy and formulation requirements for agricultural applications.

### 3.3. Rhizobial Viability on Pelleted A. sinicus Seeds: Coating Effects and Survival Assessment

Seed pelleting technology has emerged as an innovative solution to overcome the limitations of conventional microbial inoculation methods in agriculture [54,55,56]. In our study, we successfully developed uniformly coated *A. sinicus* seeds with approximately 1 mm thick pelleting layers using a rotary coating machine. The processed seeds maintained viable rhizobial populations (>100 CFU/seed) after 10 days of storage, demonstrating the technology’s potential for practical field applications. This preservation of microbial viability is particularly crucial as it ensures sufficient bacterial numbers for effective nodulation and nitrogen fixation in host plants.

The technical success of seed pelleting depends on precise optimization of multiple factors. We implemented controlled low-temperature drying at 25 °C to minimize damage to rhizobial cells during processing, with the incorporation of protective agents such as trehalose aiding in the mitigation of osmotic stress [57,58]. These measures collectively contributed to maintaining microbial viability throughout the pelleting process. Our selection of *M. huakuii* CCBAU 33470 as the inoculant strain was based on its demonstrated superior symbiotic compatibility with *A. sinicus* in preliminary screenings. The viability assessment revealed that pelleted seeds maintained substantial rhizobial populations (>1000 CFU/seed) after 15 days of storage (Figure 8A), meeting the minimum threshold of 100 CFU/seed required for effective nodulation in *A. sinicus* (under review data from personal communication). The prolonged nodulation capacity of rhizobia in pelleted seeds (up to 40 days) validated the formulation’s protective properties. These results confirm that the preserved inoculum viability is fully capable of supporting successful symbiotic nitrogen fixation.

### 3.4. Current Limitations and Future Perspectives

Looking forward, several important considerations must be addressed to translate this technology into real-world agricultural applications, because of the limitations of greenhouse experiments [59], sterilized vermiculite-based Leonard jar system compared to non-sterilized soil [60] and variable viability of rhizobia on pelleted seeds [59,61]. First, the competitive interactions between inoculated and native rhizobial strains under field conditions require systematic evaluation, as soil microbiomes and environmental variability may significantly influence nodulation efficiency. Second, the temporal dynamics of rhizobial survival in pelleted formulations, including pre-sowing storage stability and post-emergence nodulation kinetics under abiotic stresses, warrant further investigation. Additionally, long-term stability testing across diverse environmental conditions is needed to assess the robustness of this pelleting technology. Finally, the comprehensive cost–benefit analyses should be conducted to evaluate its economic feasibility for farmers, ensuring scalability and adoption potential. These future multi-faceted studies will be pivotal in determining the commercial viability and practical implementation of this seed pelleting approach.

## 4. Materials and Methods

### 4.1. Rhizobial Strains and Culture Conditions

Seven rhizobial strains originally isolated from *A. sinicus* were utilized in the current study: *Mesorhizobium huakuii* CCBAU 2609 [11], CCBAU 33470 and CCBAU 33404 [62]; and *M. jarvisii* CCBAU 33443 [62] and 7653R (originally classified as *M. huakuii*) [63]; and *M. qingshengii* CCBAU 33430 and CCBAU 33460 [2]. Among these strains, 7653R was kindly provided by Professor You-Guo Li from Huazhong Agricultural University [64], with all other strains being obtained from the CCBAU (Culture Collection of Beijing Agricultural University, Beijing, China).

The rhizobial cultures were maintained in either yeast extract–mannitol (YM) or tryptone–yeast extract (TY) medium, incubated at 28 °C with orbital shaking at 180 rpm. The YM medium was prepared with the following components (per liter): 10 g mannitol, 0.25 g K_2_HPO_4_, 0.25 g KH_2_PO_4_, 0.1 g MgSO_4_, 0.1 g NaCl, and 3.0 g yeast extract. The composition of the TY medium is as follows (g/L): Tryptone 5.0, Yeast extract 3.0, CaCl_2_ 0.6. For solid medium preparation, 15 g/L agar was added. The culture media were sterilized via autoclaving at 121 °C (0.1 MPa steam pressure) for 20 min. To enable selective cultivation and prevent bacterial contamination, nalidixic acid (NA) was added to the medium at a final concentration of 20 μg/mL. The NA stock solution was prepared following the standardized protocol of Somasegaran and Hoben [65]. Briefly, 0.2 g of NA powder was dissolved in 10 mL of sterile double-distilled water (ddH_2_O), with the addition of sodium hydroxide (NaOH) pellets to adjust the pH and enhance solubility. The solution was then filter-sterilized through a 0.2 μm membrane filter under aseptic conditions.

### 4.2. Growth Response of A. sinicus to Inoculation with Diverse Mesorhizobium Strains

Two commercially important *A. sinicus* cultivars (Yijiangzi and Luzi No. 8, gifted by Profs. Guo-Peng Zhou and Jian-Min Geng) were used for this study. Seed surface sterilization was performed sequentially using the following: 95% (*v*/*v*) ethanol for 30 s, followed by 2.5% (*w*/*v*) sodium hypochlorite solution for 2 min, with five subsequent rinses in sterile distilled water to eliminate residual disinfectants [66]. Sterilized seeds were aseptically transferred to 0.8% (*w*/*v*) water agar plates and incubated in darkness at 20 °C for 48 h to synchronize germination.

The Leonard jar assemblies were prepared following the method of Kimiti and Odee [67] with modifications, consisting of an upper plastic cup containing vermiculite moistened with 1× low-nitrogen nutrient solution (20 × stock: 0.6 g Ca(NO_3_)_2_·4H_2_O, 1.5 g KCl, 1.2 g MgSO_4_, 2.72 g K_2_HPO_4_, 9.2 g CaSO_4_·2H_2_O, and 15 g FeC_6_H_5_O_7_ per liter, supplemented with 20 mL trace element solution containing 2.86 g H_3_BO_3_, 1.81 g MnSO_4_, 0.80 g CuSO_4_·5H_2_O, 0.22 g ZnSO_4_, and 0.02 g H_2_MoO_4_ per liter) and fitted at the base with sterile absorbent gauze, connected to a lower 500 mL glass bottle filled with sterile ddH_2_O. All components were sterilized via autoclaving at 121 °C for 60 min.

Germinated seedlings exhibiting approximately 2 cm radicles were aseptically transplanted into pre-formed holes in the vermiculite medium using sterile forceps. Each seedling received 1 mL of rhizobial inoculum consisting of a cell suspension standardized to OD_600_ = 0.2 in sterile physiological saline (0.85% NaCl) prepared based on the method of Gao et al. [68]. The inoculated plants were maintained in a controlled-environment growth chamber under a 12 h photoperiod (25 °C light/23 °C dark) for 45 days, with a single supplementation of sterile water during the cultivation period to maintain optimal moisture levels.

The nodulation potential of lyophilized rhizobial formulations was evaluated using a standardized seed inoculation protocol. A precisely measured aliquot (0.1 g) of freeze-dried bacterial preparation was aseptically mixed with 10 g of surface-sterilized seeds to ensure uniform coating. The inoculated seeds were then individually transferred using sterile forceps into the previously described Leonard jar assemblies containing vermiculite growth substrate and 1 × low-nitrogen nutrient solution. All plants were cultivated under controlled environmental conditions (12 h photoperiod, 25/23 °C day/night) for 15 days to observe both vegetative growth and root nodule formation.

### 4.3. Measurement of A. sinicus Growth Traits and Nodulation Characteristics

A comprehensive growth analysis of *A. sinicus* was conducted, evaluating multiple physiological and morphological parameters. Chlorophyll content was quantified non-destructively using a SPAD-502 Plus chlorophyll meter (Konica Minolta, Osaka, Japan), with three measurements taken from the first fully expanded leaf of the primary tiller and averaged per plant. Tiller development was assessed by counting all basal shoots emerging within 2 cm of the soil surface. For biomass determination, aerial tissues were oven-dried at 60 °C for 72 h to constant weight. The fresh weight of the root nodule was measured following careful root washing to remove vermiculite particles, with all visible nodules subsequently excised using sterile forceps and immediately weighed on a precision balance (±0.1 mg).

### 4.4. Preparation of Cryoprotectant Solutions and the Freeze–Vacuum Drying Protocol

The cryoprotectant solution, consisting of different types of protectants (Table 2), was prepared, respectively, in double-distilled water (ddH_2_O) at the specified concentrations (Table 2). The solution was sterilized via autoclaving at 108 °C (0.045 MPa) for 15 min [49]. *Mesorhizobium huakuii* CCBAU 33470 was used for freeze-drying preparation. A single colony of strain CCBAU 33470 grown on a YM agar (YMA) solid plate was aseptically transferred into 5 mL of YMA liquid medium using a sterile pipette and incubated at 28 °C with 180 rpm agitation for 72 h to obtain the primary culture. This starter culture was then inoculated into 500 mL of fresh YMA medium at an initial OD_600_ of 0.02 and grown under identical conditions for 96 h. Bacterial cells were harvested via centrifugation at 6000× *g* for 5 min at 4 °C, followed by supernatant removal. An equal volume of cryoprotectant solution was added to the cell pellet for resuspension. After thorough mixing with a sterile pipette tip, the suspension was pre-frozen in a −20 °C freezer for 4 h, followed by freezing in a −80 °C freezer for 12 h. The samples were then freeze-dried (−55 °C) for 6 h using a freeze–vacuum dryer (Labconco, Kansas, MO, USA).

Under sterile conditions in a laminar flow hood, the lyophilized samples were aseptically transferred into 5 mL sterile glass vials (sealed with rubber stoppers) using flame-sterilized forceps. To ensure anaerobic storage conditions, the headspace air was evacuated from each vial using a sterile 5 mL syringe. Following evacuation, both the needle puncture site and the vial opening were securely sealed with Parafilm^®^ to maintain sample integrity. Processed vials were subsequently stored at 4 °C for long-term preservation.

### 4.5. Rhizobial Cell Counting (CFU Enumeration)

The lyophilized rhizobial cells were reconstituted in YMA liquid medium supplemented with 1.5% (*w*/*v*) trehalose at a 1:19 (*v*/*v*) ratio, achieving a final 20-fold dilution. Using aseptic technique, a 200 μL aliquot of the reconstituted suspension was mixed with 800 μL of sterile distilled water to generate a 10^−2^ dilution. Subsequent decimal dilutions (10^−6^ to 10^−8^) were prepared through serial dilution in sterile water for viable cell counting.

For viable cell enumeration, 100 µL aliquots from each dilution (10^−6^ to 10^−8^) were aseptically spread-plated onto YMA in triplicate. Following inverted incubation at 28 °C for 6–7 days, plates containing 30–300 discrete colonies were selected for counting. The colony-forming units (CFUs) per milliliter were calculated according to the following formula:

CFU/mL = (number of colonies × dilution factor)/volume plated (mL)

### 4.6. Seed Coating and Rhizobial Viability Assessment

The seed coating procedure was performed using a commercial seed coating machine (Model KRT-300, Hebei Kairuote Machinery Manufacturing Co., Ltd., Baoding, Hebei, China) following the manufacturer’s protocol. During seed coating, log-phase cultures of actively growing *M. huakuii* CCBAU 33470 were incorporated into the coating matrix (purchased from the same manufacturer as the seed coating machine) and dried under forced air at 25 °C. The coated seeds were subsequently vacuum-sealed in moisture-proof packaging and stored at 4 °C to maintain rhizobial viability.

For quantification of surface-adhered rhizobia, 100 coated seeds were vortexed in 20 mL of yeast–mannitol (YM) broth at 180 rpm for 15 min to dislodge bacterial cells. The resulting suspension was subjected to serial decimal dilution in YM broth, followed by spread-plating on YM agar. Colony-forming units (CFUs) were enumerated after 5–7 days of incubation at 28 °C, with the results expressed as viable rhizobial cells per seed (CFU/seed). Seed pellets that had been stored for 40 days were sown in triplicate into Leonard jars containing vermiculite and a 1× low-nitrogen nutrient solution. Plants were maintained under controlled greenhouse conditions as previously described, and nodule formation was evaluated 15 days post-sowing.

### 4.7. Statistical Analysis

The data for each variable were rigorously analyzed to assess variability and significance. Variance analysis was performed using one-way analysis of variance (ANOVA) in GraphPad Prism 9.5 and IBM SPSS Statistics 27 software, followed by Tukey’s honestly significant difference (HSD) test and the least significant difference (LSD) test for post hoc multiple comparisons. For datasets violating the assumptions of normality, the non-parametric Kruskal–Wallis test was employed. Data visualization and graphical representations were generated using GraphPad Prism 9.5. To ensure robust statistical power and reliable results, each *A. sinicus* rhizobium inoculation treatment was analyzed with a minimum of eight biological replicates.

## 5. Conclusions

The results of this study demonstrate that *Mesorhizobium huakuii* CCBAU 33470 exhibits a superior symbiotic compatibility with *Astragalus sinicus*, with lyophilized formulations (20% trehalose or skimmed milk powder) maintaining a high viability (>10^10^ CFU/g) for two months and significantly enhancing nodulation, tiller production, and biomass. The developed seed pelleting technique retained >10^3^ CFU/seed after 15 days of storage, overcoming key limitations in inoculant application and strain availability while providing a transferable model for small-seeded legumes such as *Medicago sativa* and *Trifolium* spp. This integrated approach—combining elite rhizobial strain selection, optimized preservation, and efficient delivery—represents a significant advancement in leguminous green manure-based cultivation systems, particularly for rice rotation as well as orchard and tea plantation systems [69].

## Figures and Tables

**Table 1:** Viable cell counts of rhizobia after freeze–vacuum drying with different cryoprotectants.

Cryoprotectants	Present (%)	lg (CFU/g)	Cryoprotectants	Present (%)	lg (CFU/g)
Trehalose	15	11.04 ± 0.04	Sorbitol	3	/
	20	11.55 ± 0.02		5	11.73 ± 0.02
	25	11.27 ± 0.05		7	11.67 ± 0.04
Gelatin	1	9.80 ± 0.08	Betaine	1	9.76 ± 0.14
	2	/		2	11.62 ± 0.02
	3	11.74 ± 0.03		3	10.03 ± 0.09
Skimmed milk powder	15	11.51 ± 0.06	Polyvinylpyrrolidone K30	7	10.94 ± 0.02
	20	11.58 ± 0.07		10	9.75 ± 0.07
	25	11.31 ± 0.01		13	/

Note: A slant (/) indicates that no single colonies were detected at the same dilution.

**Table 2:** Performance assessment of cryoprotectants in maintaining *M. huakuii* CCBAU 33470 cell viability post-lyophilization.

Protectant	Mass Fraction (*w*/*v*, %)		
Trehalose	15	20	25
Skimmed milk powder	15	20	25
Gelatin	1	2	3
Betaine	1	2	3
Sorbitol	3	5	7
Polyvinylpyrrolidone K30	7	10	13

## Data Availability

No new data were created.

## Supplementary Materials

The following supporting information can be downloaded at https://www.mdpi.com/article/10.3390/plants14152431/s1, Figure S1: Growth status of Luzi No. 8 plants 45 days after inoculation with different rhizobia. (A) CCBAU 33404, (B) CCBAU 33470, (C) CK, (D) 7653R, and (E) CCBAU 33460; Figure S2: Growth status of Yijiangzi plants 45 days after inoculation with different rhizobia. (A) CCBAU 33404, (B) CCBAU 33470, (C) CK, (D) 7653R, and (E) CCBAU 33460. Figure S3: Growth status of Yijiangzi plants 45 days after inoculation with different rhizobia. (A) CCBAU 33430, (B) CCBAU 33460, (C) CCBAU 33443, (D) CCBAU 33470, (E) CK, (F) CCBAU 2609, (G) 7653R.

## Abbreviations

The following abbreviations are used in this manuscript:

CCBAU	Culture Collection of Beijing Agricultural University, Beijing, China
CK	Control
PVP	Polyvinylpyrrolidone
CFU	Colony-forming Unit
Smp_20_	20% skimmed milk powder
Tre_20_	20% trehalose
*M.*	*Mesorhizobium*
*M. huakuii*	*Mesorhizobium huakuii*
*A. sinicus*	*Astragalus sinicus*
DMSO	Dimethyl sulfoxide
YM	Yeast extract–mannitol medium
TY	Tryptone–yeast extract medium
YMA	Yeast–extract mannitol agar medium
rpm	Rotation per minute
NA	Nalidixic acid
OD_600_	Optical density at 600 nm
