# Supplementary material for: Screening and Application of Highly Efficient Rhizobia for Leguminous Green Manure Astragalus sinicus in Lyophilized Inoculants and Seed Coating
Source: Plants (Basel). 2025 Aug 6;14(15):2431. doi: 10.3390/plants14152431 (PMC12349254; doi:10.3390/plants14152431)
Supplement: Supplementary file 1 [file plants-14-02431-s001.zip › plants-3729715-supplementary.pdf]

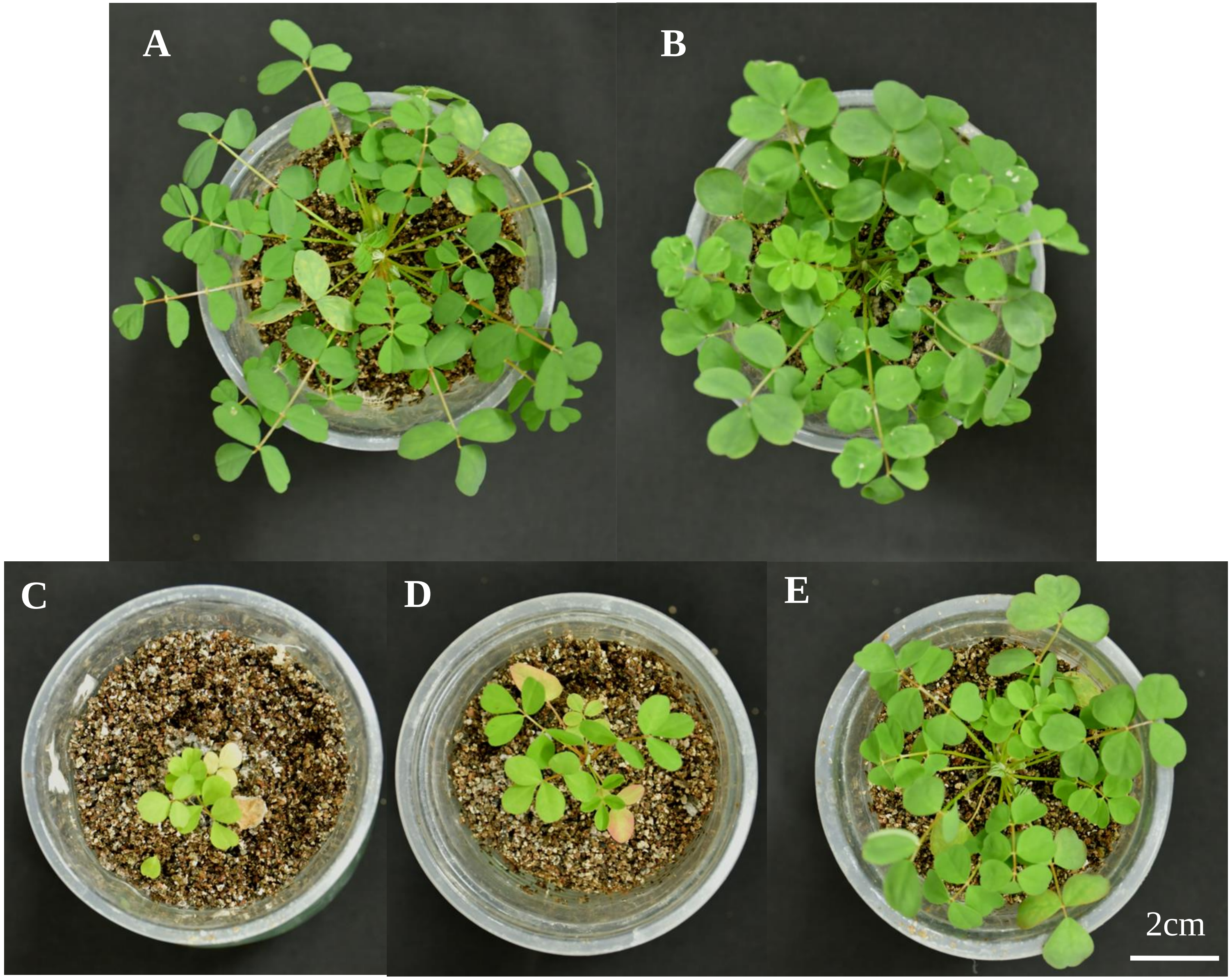

Supplementary Figure S1. Growth status of Luzhi No.8 plants 45 days after inoculation with different rhizobia. (A) CCBAU 33404, (B) CCBAU 33470, (C) CK, (D) 7653R, (E) CCBAU 33460.

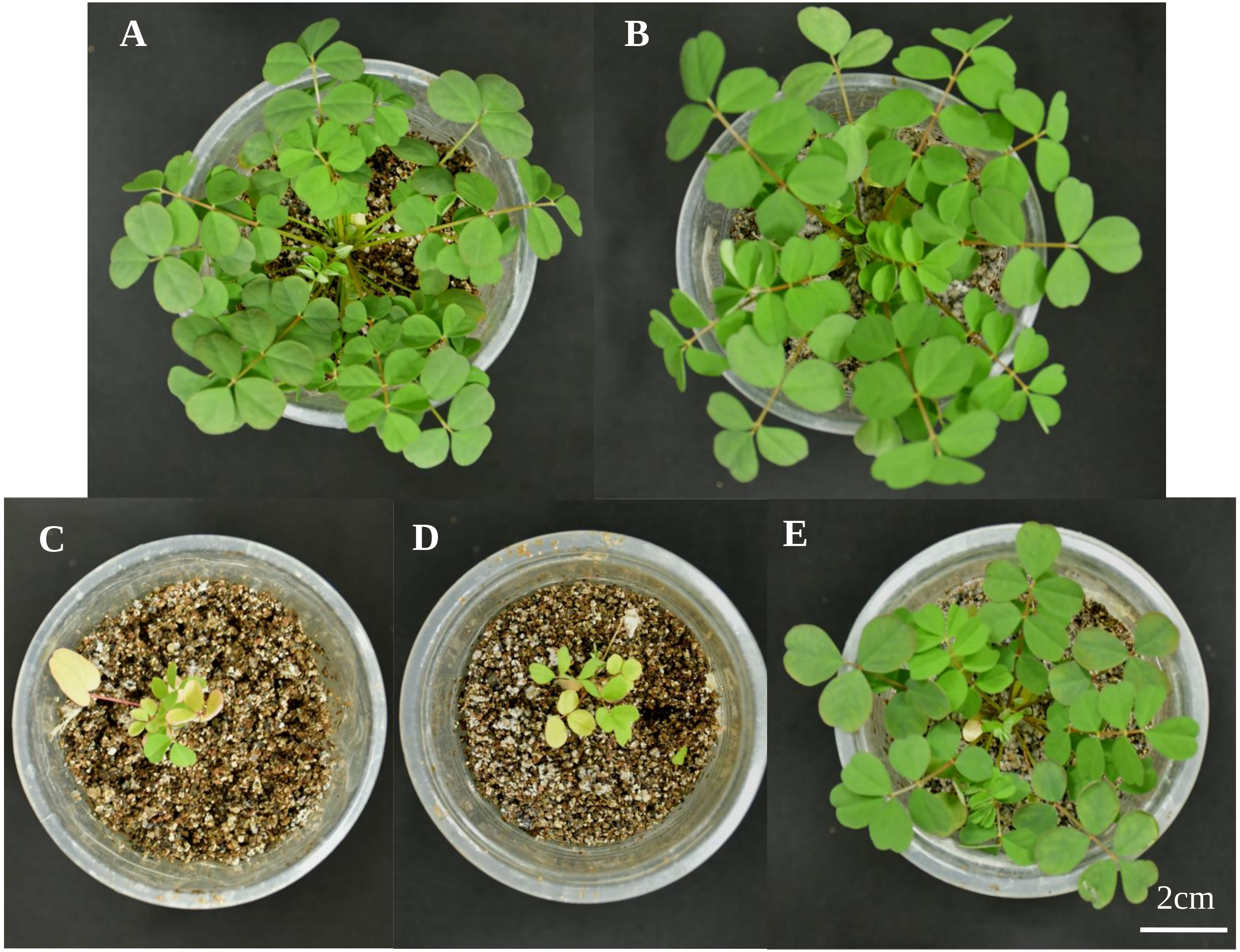

Supplementary Figure S2. Growth status of Yijiangzi plants 45 days after inoculation with different rhizobia. (A) CCBAU 33404, (B) CCBAU 33470, (C) CK, (D) 7653R, (E) CCBAU 33460.

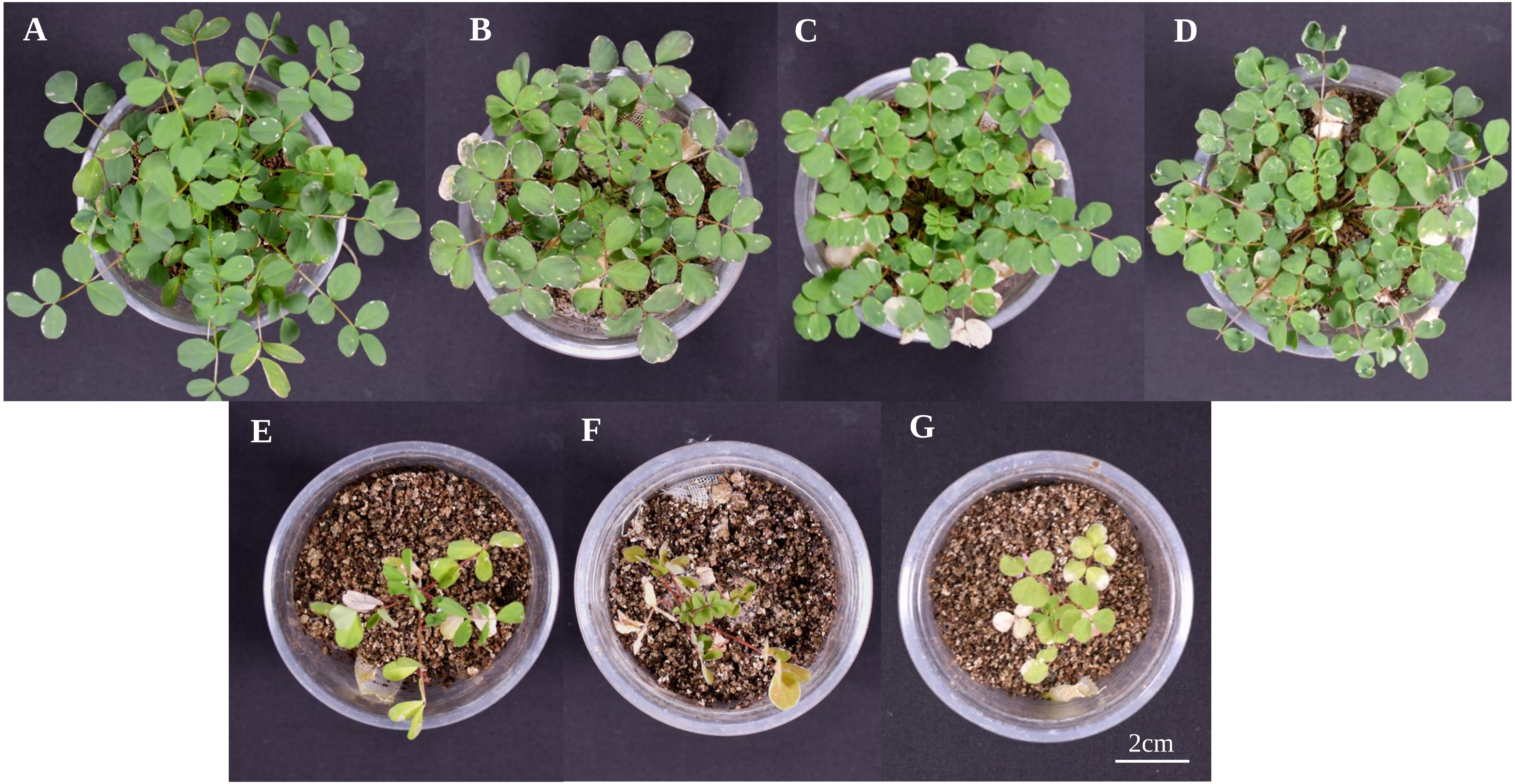

Supplementary Figure S3. Growth status of Yijiangzi plants 45 days after inoculation with different rhizobia. (A) CCBAU 33430, (B) CCBAU 33460, (C) CCBAU 33443, (D) CCBAU 33470, (E) CK, (F) CCBAU 2609, (G) 7653R.
